# Compressive Strength of Mineral Trioxide Aggregate with Propylene Glycol

**DOI:** 10.22037/iej.2016.13

**Published:** 2016

**Authors:** Negin Ghasemi, Saeed Rahimi, Shahriar Shahi, Amin Salem Milani, Yashar Rezaei, Mahnaz Nobakht

**Affiliations:** a *Dental and Periodontal Research Center, Department of Endodontics, Dental School, Tabriz University of Medical Sciences, Tabriz, Iran; *; b * Department of Endodontics, Dental School, Tabriz University of Medical Sciences, Tabriz, Iran; *; c * Department of Dental Biomaterials, Dental School, Tabriz University of Medical Sciences, Tabriz, Iran; *; d * Dental School, Tabriz University of Medical Sciences, Tabriz, Iran*

**Keywords:** Compressive Strength, Mineral Trioxide Aggregate, Propylene Glycol

## Abstract

**Introduction:**

The aim of this study was to evaluate the effect of adding propylene glycol (PG) to mineral trioxide aggregate (MTA) liquid with volume ratio of 20% on the compressive strength (CS) of MTA in two time periods (4 and 21 days) after mixing.

**Methods and Materials:**

Four groups of steel cylinders (*n*=15) with an internal diameter of 3 and a height of 6 mm were prepared and MTA (groups 1 and 2) and MTA+PG (80% MTA liquid+20% PG) (groups 3 and 4) were placed in to the cylinders. In groups 1 and 3 the CS was evaluated after 4 days and in groups 2 and 4 after 21 days. Data were calculated using the two-ways ANOVA. The level of significance was set at 0.05.

**Results:**

The highest (52.22±18.92 MPa) and lowest (4.5±0.67 MPa) of CS was obtained in 21-day MTA samples and 4-day MTA+PG specimen, respectively. The effect of time and PG were significant on the CS (*P*<0.05). Mixing MTA with PG significantly reduced the CS; but passing the time from 4 to 21 days significantly increased the CS.

**Conclusion:**

Considering the limitations of this study, PG had a negative effect on CS of MTA.

## Introduction

Mineral trioxide aggregate (MTA) is a calcium silicate-based hydrophilic cement consisting of powdered particles that are composed of Portland cement, gypsum and bismuth oxide which sets in contact with the liquid [1, 2]. Due to having several favorable characteristics, MTA has variable applications in endodontics such as perforation repair and root canal treatment of immature teeth; but long setting time and difficult handling are among its disadvantages [3].

Several strategies have been proposed to improve the properties of the MTA, one of which is to add propylene glycol (PG) to MTA liquid [4-6]. PG is a nontoxic alcoholic viscose composition that is safely used in food and pharmaceutical compounds [7, 8]. Various studies evaluated the properties of MTA by adding PG. According to the results of these studies, PG improves the consistency and handling, push out bond strength and reduces the setting time. It also increases pH and the release of calcium hydroxide in the initial phase of setting [4-6, 8-13]. Adding PG has no negative effect on the biocompatibility and the biological response of cells to MTA is similar in the presence or absence of PG [9].The microhardness and hardness of MTA is reduced by the addition of PG [7]. 

Considering the clinical applications of MTA, compressive strength (CS) of this material, is important in some cases; for example when MTA is used to repair furcal perforations where it must tolerate occlusal forces and the placement of restorative material on it [14, 15]. CS of hydraulic cements is as an indicator of hydration reaction and in fact, indirectly is a reflex of the setting process of the material. These physical properties are affected by the type of MTA, the mixing liquid, condensing pressure and mixing techniques used to mix the powder and liquid [15-18].

Studies conducted on the CS of MTA, indicate that the hydration catalysts such as calcium chloride, etching, the presence of blood contamination, fetal bovine serum (FBS), increased ratio of liquid to powder, all can decrease the CS [12, 15, 19-22]. However, addition of silica nanoparticles and bismuth oxide have no effect on it [23], but mixing with 0.12% chlorhexidine and mechanically mixing using amalgamators and placement with ultrasonic agitation increase the CS of MTA [14-17].

So far, there have been no published studies on the effect of PG on the CS of MTA. This *in vitro *study aimed to evaluate the effect of adding PG into MTA liquid (with volume ratio of 20%) on the CS of MTA in two time periods of 4 and 21 days after mixing. The null hypothesis in this study was that PG has no effect on the CS.

## Materials and Methods

Based on the used material and the assessment time of CS, 60 samples were assigned into 4 groups in this study (*n*=15). Fifteen steel cylinders with an internal diameter of 3 mm and height of 6 mm were prepared for each group. In groups 1 and 2 the cylinders were filled with Angelus MTA (Angelus, Londrina, Paraná, Brazil) and in groups 3 and 4 they were filled with MTA mixed with 0.2 mL PG (Merk, Darmstadt, Germany) and 0.8 mL MTA liquid. In the other words, 4 volumes of MTA liquid were mixed with one volume of PG. The volumes were measured using insulin syringe.

CS of the samples was measured using ISO 6876 guidelines [14, 15, 17]. Materials and instruments, before being used in this study, were kept at 23±1^°^C for 1 h. All samples were mixed by one operator and mixing time was 10 sec for all samples. Within 2 min after initiation of mixing, the materials were placed into steel cylinders and were packed with an appropriate sized condenser. The internal surfaces of the cylinders were greased using paraffin, before material placement. Then the samples were wrapped in gauze soaked in distilled water and were kept in a closed container at 37^°^C until the CS was measured. 

CS assessment was performed using universal testing machine (Hounsfield Test Equipment, model: H5K-S, Perrywood Business Park, Honey Corckland, Salfords, Redhill, Surrey, UK) 4 (groups 1 and 3) and 21 (groups 2 and 4) days after mixing and placement of the materials in to the cylinders. Cross head of the device applied force at a speed of 1 mm/min in the direction parallel to the longitudinal axis of the molds until the materials were crushed. This force was recorded based on Newton and was converted into MPa using the following formula: CS=4*p*/*μd*2 where *p* is the maximum force applied in Newton's, and *d* is the mean diameter of the specimen in mm.

Data were analyzed using the descriptive statistics (mean±SD). The Kolmogorov-Smirnov test was used to evaluate normal distribution of data and the Levene's test was applied for equality of error variances. Considering the normal distribution of data (*P*=0.3) and equality of the variances (*P*=0.06), the two-way ANOVA was used to evaluate the significant effect of the material type and time period and also for the two-by-two comparison of the groups. SPSS software (SPSS version 18.0, SPSS, Chicago, IL, USA) was used for the analysis of data. The level of statistical significance was defined at 0.05.

## Results

The highest (52.22±18.92 MPa) and lowest (4.5±0.67 MPa) values of CS was obtained in the second (MTA/21 days) and third (MTA+PG/4 days) groups, respectively. 

The ANOVA analysis showed a significant effect of the presence or absence of PG and time span on the CS (*P*=0.02 and *P*=0.001, respectively). The presence of the PG significantly reduced the strength (*P*<0.05). Regardless of the presence or absence of PG, the passage of time from 4 to 21 days, significantly increased the CS (*P*=0.03 for MTA groups and *P*=0.0001 for MTA+PG groups). Pairwise comparison of the studied groups showed significant differences between all groups (*P*<0.05). 

## Discussion

This study aimed to assess the effect of PG on the CS of MTA. In addition to the presence or absence of PG which was added with volume ratio of 20% to MTA liquid, another variable of the study was to evaluate the effect of time on the CS which was considered 4 and 21 days after mixing. The null hypothesis was rejected and PG significantly reduced the CS. Over time, the CS of all groups in the study was increased.

MTA is a calcium silicate material and with regard to its applications in the field of root canal treatment [2], the CS has always been of high importance in various studies [15]. The initial CS of MTA is 40 MPa according to previous conducted studies that reaches to 67 MPa after 21 days [17]. CS indicates the quality of hydration process and the hydration is a factor that directly affects the MTA setting; therefore, any factor that affects the hydration process is effective on the physical properties [14]. PG is an alcoholic compound that is added to MTA to improve its properties. Previous studies on some of the physical properties of MTA such as flow, pH, setting time and the release of calcium hydroxide, have proved its desired properties [6-8]. 

**Table 1:** Mean (SD) of the CS of study groups in MPa

**Material **	**Time **	**CS**
**MTA**	4 days	35.85 (12.34)
	21 days	51.22 (18.92)
**MTA+PG**	4 days	4.5 (0.67)
	21 days	16 (6.78)

In this study, we chose two time periods to assess the CS. The shorter time span of 4 days was selected due to the fact that the primary strength is important in clinical applications because initially, the material is exposed to occlusal forces in the patient's mouth. Also in this period, the material will reach to optimum setting and also in the previous studies, the same time period was taken into consideration for the same purpose [14, 15, 17]. Time period of 21 days was chosen in order to investigate the effect of PG, in the longer time period, besides in the short time and compare the results with control group (without PG). Long-term strength is important for the material resistance against crushing caused by forces resulted from placing restorative materials and occlusal forces [17]. In this study, PG with volume ratio of 20% was added into MTA liquid, which according to previous studies, has the best effect on pH and the release of calcium hydroxide and setting time and has minimal negative effect on its micro hardness [7, 8].

In this study, attempts were made to homogenize factors affecting the physical properties of the MTA [14, 15, 17]. The powder to liquid ratio was similar in all samples (3:1). Mixing the powder in the liquid was done manually by one person for all samples for 10 sec. Materials were later placed into the cylinder by one operator using a condenser with a selected unit size in order to homogenize the condensed forces. In previous studies, the mechanical mixing and placement with ultrasonic agitation increased the CS [14, 15]. But in this study, we used only the manual method to remove the interaction between adding PG and mixing and placement method, because it was the first time that addition of PG and its effect on the compressive strength were investigated. Temperature and humidity were kept the same for all samples.

In our study, by adding PG to MTA liquid, the CS significantly decreased. PG is a hygroscopic compound and reduces the amount of water available for hydration and MTA setting and affects the nucleation of calcium silicate hydrate, which is the possible reason for the result obtained in this study. The effect of PG on the strength of MTA is similar to the effect of hydration accelerators such as calcium chloride since those materials reduce the CS, as well [21]. Increased amount of CS over time in both groups in our study is in agreement with results of previous studies [16, 17]. 

According to our results and the results of previous conducted studies, properties on which PG has a negative effect are CS, microhardness and hardness of MTA [7]; which seems to be due to the reason that all these properties are directly dependent on hydration of powdered particles which is disrupted by addition of PG.

Although, we have tried to minimize the effect of confounding variables in the study; however, we should still consider the clinical application of this material in interpreting and generalizing the results of the study too. Because a series of factors with which the material is faced in the clinical application, are not included in the study, such as the presence of blood contamination in the area, the presence of an acidic environment due to inflammation, bacterial products, interstitial fluid elements, *etc*.

## Conclusion

Under the limitations of this study, the use of PG for mixing MTA is not recommended where the CS is important, such as repair of furcal perforation; because the material must be resistant against being crushed as a result of occlusal forces and the placement of restorative material on it. However, given that PG significantly improves other properties of MTA, it is recommended to use it as apical plug and retro filling material in surgery in cases where the CS is not important.
